# Regulation of T cell repertoires by commensal microbiota

**DOI:** 10.3389/fcimb.2022.1004339

**Published:** 2022-10-13

**Authors:** Kwang Soon Kim

**Affiliations:** Department of Life Sciences, Pohang University of Science and Technology (POSTECH), Pohang, South Korea

**Keywords:** gut microbiota, T cell repertoire, antigenic mimicry, autoimmune disease, anti-tumor therapy

## Abstract

The gut microbiota plays an important role in regulating the host immune systems. It is well established that various commensal microbial species can induce the differentiation of CD4^+^ T helper subsets such as Foxp3^+^ regulatory T (Treg) cells and Th17 cells in antigen-dependent manner. The ability of certain microbial species to induce either Treg cells or Th17 cells is often linked to the altered susceptibility to certain immune disorders that are provoked by aberrant T cell response against self-antigens. These findings raise an important question as to how gut microbiota can regulate T cell repertoire and the activation of autoreactive T cells. This review will highlight microbiota-dependent regulation of thymic T cell development, maintenance of T cell repertoire in the secondary lymphoid tissues and the intestine, and microbiota-mediated modulation of autoreactive and tumor neoantigen-specific T cells in autoimmune diseases and tumors, respectively.

## Introduction

T cell repertoires with the high levels of T cell receptor (TCR) diversity are a key determinant for the host’s ability to defend itself against numerous environmental pathogens (Nikolich-Zugich et al., 2004). T cell repertoires can be shaped at multiple levels (Klein et al., 2014). During thymic T cell development, random rearrangement of TCR gene segments and combination of TCRα and TCRβ chains create enormous levels of TCR diversity. Negative selection of developing T cells in the thymus restricts TCR repertoires and mainly prevent the release of naïve T cells with strong affinity to self-antigens (self-Ags), of which activation can be potentially harmful, resulting in autoimmune diseases. Furthermore, developing T cells with higher affinity to self-Ags can have alternative cell fates and differentiate into Foxp3^+^ regulatory CD4^+^ T (Treg) cells (Hsieh et al., 2012). Such thymic Treg cells play an important role in suppressing the activation of autoreactive T cells that escape from negative selection in the thymus. Maintenance of T cell repertoires in the periphery is also affected by the affinity to self-peptide MHC I or II complexes and the exposure to homeostatic cytokines such as IL-7 and IL-15 (Surh and Sprent, 2008). Even in the steady state, upon the exposure to innocuous Ags such as commensal microbiota and dietary Ags through mucosal tissues such as gastrointestinal tract, naïve T cells can differentiate into effector or tissue-resident T cells, of which maintenance is distinct from that of naïve T cells.

**Figure 1:**
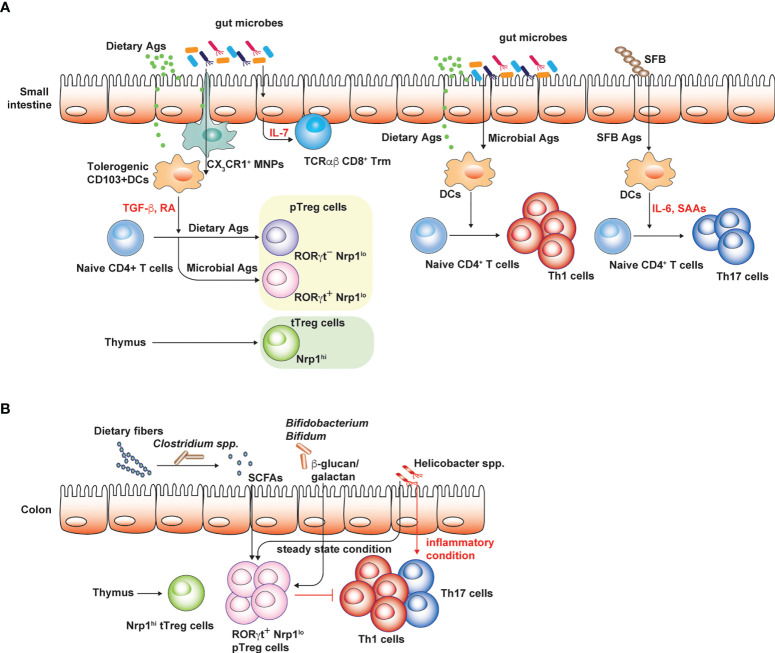
Conventional T cell subsets in small intestine and colon. Regulatory and pro-inflammatory T cell responses are well-balanced to tightly control overt immune responses against both dietary and microbial Ags. **(A)** CD103^+^ DCs in the small intestine are typically tolerogenic and express TGF-β and retinoic acid (RA), which promote peripheral Treg cell generation. Small intestinal Treg cells consist of thymic Treg cells specific to self-Ags or presumably microbial Ags, the latter delivered into the thymus by CX3CR1 mononuclear phagocytic cells in early-life. RORγt^+^ Nrp1^lo^ pTreg cells are thought to be induced by gut microbiota while RORγt^-^ Nrp1^lo^ pTreg cells are generated by dietary Ags. These intestinal Treg cells limit intestinal proinflammatory Th1 and Th17 cell responses against dietary Ags and gut microbiota. IL-7 produced by epithelial cells in gut microbiota-dependent manner promotes the survival and maintenance of tissue resident TCRαβ CD8^+^ T cells. **(B)** Colonic Treg cells largely consist of RORγt^+^ pTreg cells and thymic Treg cells. Broad spectrum of microbes can induce pTreg cells through various mechanisms. Under steady state condition, colonic Th17 cells are rare relative to Th1 cells and certain pathobiont species such as Helicobacter spp. promote Treg cell generation. However, in inflammatory settings, these pathobiont species effectively induce colonic Th17 cell responses. The figure was created with Adobe illustrator.

Commensal microbiota, the complex community of microbial species especially in mucosal tissues, such as respiratory, gastrointestinal and urogenital tract, can shape tissue-specific T cell responses. It is well known that gut microbiota induce *de novo* differentiation of CD4^+^ T cells into various T helper subsets such as peripheral Foxp3^+^ Treg (pTreg) and Th17 cells in the intestine (**Figure 1**). pTreg cells generated by the gut microbiota prevent overt immune responses against Ags derived from diet and gut microbes, endorsing tonic or well-balanced proinflammatory T cell responses that can be helpful for the protection against intestinal pathogens (Tanoue et al., 2016). Th17 cells that are abundantly found in the small intestine are generated by certain microbial species such as segmented filamentous bacteria or *Bifidobacterium adolescentis* (Ivanov et al., 2009; Tan et al., 2016) (**Figure 1A**). These Th17 cells generated in responses to gut microbes enhance the intestinal barrier functions, thereby mediating the protection against pathogenic fungal infection (Ivanov et al., 2009). However, Th17 cell responses in the small intestine are strongly associated with the host susceptibility to experimental autoimmune encephalomyelitis (EAE) used as an animal model for multiple sclerosis, a Th17-mediated autoimmune disease in human. Therefore, germ-free (GF) mice with markedly reduced levels of intestinal Th17 cells due to the absence of gut microbiota are resistant to EAE (Berer et al., 2011; Lee et al., 2011). Furthermore, the transfer of gut microbiota from multiple sclerosis patients into GF spontaneous EAE models increases the incidence of CNS autoimmunity (Berer et al., 2017). Considering that EAE is mediated by Th17 cells in the CNS or spinal cords that are specific to myelin sheath proteins (Berer et al., 2011), it has been enigmatic as to how the gut microbiota promotes pathogenic Th17 responses against self-Ags. In addition, the dysbiosis of the gut microbiota is considered as an important factor that contributes to the higher incidence of autoimmune diseases (Miyauchi et al., 2022).

As exemplified by the influence of the gut microbiota on the autoimmune disease, gut microbiota profoundly shapes T cell responses against tumor neoantigens and determines the responsiveness of tumor patients to immune-checkpoint inhibitor treatment (Sepich-Poore et al., 2021). Utilization of TCR-transgenic (Tg) cells specific to microbial Ags, peptide-loaded MHC tetramer (pMHC tetramer)-based examination of microbial Ag-specific T cells, and TCR repertoire analysis based on bulk or single cell-based TCR sequencing can improve our understanding on how the gut microbiota influences the disease pathogenesis. These mechanisms are possible through gut microbial regulation of TCR repertoires and their influences on T cell responses against self-Ags or tumor neoantigens that are previously thought to be irrelevant to the gut microbiota.

## Regulation of innate and conventional T cell development in the thymus by gut microbiota

During the T cell development in the thymus, developing T cells undergo positive and negative selection that prevent the release of T cells with defective TCR and autoreactive T cells into the periphery. Pre-thymic T cell precursors originate from common lymphoid progenitors in the bone marrow and undergo four double negative (DN) stages (from DN1 or early thymic progenitor to DN4) (Hosokawa and Rothenberg, 2021). Early thymic progenitors or thymocytes in the early DN2 stage are not fully committed to conventional T cells and can differentiate into γδ T cells. T cells that undergo through TCRβ gene rearrangement develop into double positive (DP) thymocytes upon the TCRα gene rearrangement. DP thymocytes that fail to recognize peptide-MHC (pMHC) complex expressed on cortex epithelial cells (cTECs) in the thymus undergo apoptosis (death by neglect). Meanwhile, DP thymocytes that successfully recognize pMHC complex can survive (positive selection) and pass through the next selection process (negative selection). Single positive (SP) thymocytes that express either CD4 or CD8 coreceptor migrate into the medulla region of the thymus where negative selection occurs. Particularly, medullary thymic epithelial cells (mTECs) express tissue-associated Ags in the context of MHC I and II complex through the action of Aire and Fezf2, inducing the apoptosis of T cells that recognize tissue-associated Ags with high affinity or the development of thymic Treg cells (Takaba et al., 2015; Anderson and Su, 2016).

In contrast to the development of conventional T cells, thymic Treg cells and unconventional T cells such as invariant Natural Killer T (iNKT) cells and Mucosal-Associated Invariant T (MAIT) cells are mostly developed from DP thymocytes by agonist selection and escape from cell death during negative selection (Bendelac et al., 2007; Legoux et al., 2020). DP thymocytes committed to iNKT and MAIT cells have restricted TCR usages that recognize glycolipid or vitamin B2 (riboflavin) metabolites loaded onto CD1d and MR1, respectively. Previously, it was reported that microbiota or microbial metabolites influence the thymic development of innate lymphocytes. These innate lymphocytes typically express promyelocytic leukemia zinc finger (PLZF) that is encoded by the gene *Zbtb16* and includes iNKT cells and MAIT cells. GF mice display reduced levels of PLZF^+^ innate lymphocytes in the thymus relative to conventional specific pathogen-free (SPF) mice (Ennamorati et al., 2020). Impaired development of thymic innate lymphocytes in GF mice is caused by the absence of TCR signaling due to the absence of microbial ligands, which can travel from mucosal tissues to the thymus (Legoux et al., 2020). For example, colonization of riboflavin-synthesizing microbes, but not microbes with defective vitamin B2 metabolic pathway, can restore MAIT cell development in the thymus (Legoux et al., 2019). Administration of MR1 ligands such as 5-2(2-oxopropylideneamino)-6-D-ribitylaminouracil (5-OP-RU) can induce the expansion of MAIT cells in the thymus (Legoux et al., 2019). Influences of entero-thymic communication on the development of thymic innate lymphocytes are highly dependent on the specific developmental window. In particular, early-life is critical for the development of PLZF^+^ innate lymphocytes. Early-life antibiotics treatment, but not antibiotics treatment during adulthood, can lead to permanent impairment of PLZF^+^ innate lymphocytes in the thymus (Ennamorati et al., 2020).

As seen by the influence of microbiota in thymic development of innate lymphocyte, conventional T cell development in the thymus is also affected by the gut microbiota [for a detailed review of the role of gut microbiota in T cell development, see Hebbandi Nanjundappa et al. (2022)]. mTECs express various pattern recognition receptors (PRRs) such as Toll-like receptors (TLRs) and intracellular receptors, such as nucleotide-binding oligomerization domain-containing protein 1 (NOD1) (Nakajima et al., 2014; Voboril et al., 2020). It was shown that TLR/Myeloid differentiation factor 88 (MyD88)-dependent signaling is required for the induction of functional thymic Treg cells. TLR/Myd88-dependent signaling in mTECs promote the recruitment of CD14^+^ monocyte-derived dendritic cells, which acquire mTEC-derived tissue-associated Ags and induce thymic Treg cells. However, TLR/MyD88-dependent recruitment of CD14^+^ dendritic cells is mediated by signals of endogenous origin but not exogenous TLR signals produced by gut microbiota (Voboril et al., 2020). In contrast, Aire expression in mTECs is lower in GF mice than in wild type SPF mice, suggesting that gut microbiota contributes to mTEC-mediated clonal selection of T cells (Nakajima et al., 2014). Recently, pMHC tetramer staining of segmented filamentous bacteria (SFB)-specific T cells reveals that SFB colonization can lead to an expansion of SFB-specific CD4^+^ T cells in the thymus (Zegarra-Ruiz et al., 2021). Entero-thymic communication that results in the expansion of SFB-specific CD4^+^ T cells by delivering SFB Ags is mediated by CX3CR1^+^ monocyte-derived cells, but not CD103^+^ dendritic cells, which travel from the intestine to the thymus. Interestingly, the convoy of microbial Ags by CX3CR1^+^ cells is developmentally regulated and occurs only during early-life, since the thymic expression of CCR5 ligands and CX3CL1 decreases in adult mice compared to young mice (Zegarra-Ruiz et al., 2021). However, the role of microbial Ag presentation by CX3CR1^+^ cells in the clonal selection of developing T cells and the fate of thymic-origin microbe-specific T cells in the periphery, i.e., the development of microbe-specific Foxp3^+^ regulatory CD4^+^ T cells, remains elusive.

## Regulation of T cell repertoire maintenance in the periphery by gut microbiota

Maintenance of peripheral naïve T cells requires tonic TCR signaling from self-pMHC complex and homeostatic cytokines, especially, IL-7 (Surh and Sprent, 2008). The strength of TCR signaling which is correlated with the surface expression levels of CD5 governs the responsiveness of naïve CD8^+^ T cells to foreign Ags and to cytokines such as IL-2, IL-15 and type I IFNs (Cho et al., 2010; Ju et al., 2021). IL-7 signaling is critical not only for the development of conventional T cells and innate lymphoid cells (ILCs) in the thymus but also for B cell development in the bone marrow (Barata et al., 2019). Opposite to the disposable role of IL-7 signaling in the maintenance of peripheral B cells, IL-7 signaling is important for the maintenance of peripheral naïve T cells as well as ILCs which express IL-7Rα (Martin et al., 2017; Yang et al., 2018). IL-7 is produced constitutively by stromal cells such as fibroblastic reticular cells, endothelial cells in the secondary lymphoid tissues, medullary and cortical thymic epithelial cells, VCAM1^+^ stromal cells in the bone marrow, and hepatocytes (Barata et al., 2019). IL-7 signaling through its receptor (heterodimer consisting of IL-7Rα and common γ chain) promotes T cell survival by inducing the expression of anti-apoptotic molecules such as Bcl-2 and Mcl-1 (Opferman et al., 2003; Wojciechowski et al., 2007). Interestingly, T cell populations responding to IL-7 can downregulate the surface expression of IL-7Rα (Kerdiles et al., 2009), thereby increasing the availability to other immune cell populations that requires IL-7 for their survival. However, ILCs that are outnumbered by T cells do not downregulate IL-7Rα expression (Martin et al., 2017). In this regard, ILCs can be maintained by outcompeting IL-7 with conventional T cells. Depletion of ILCs can enhance the availability of IL-7 to conventional T cells.

It has been examined whether gut microbiota can influence the signals, such as IL-7, required for maintenance of peripheral T cells or not. Homeostatic T cell proliferation was examined in SPF and GF lymphpenic *Rag1^-/-^* mice reconstituted with TCR-transgenic (TCR-Tg) T cells, such as ovalbumin-specific OT-I CD8^+^ T cells, without cognate Ag treatment (Kim et al., 2018). In this experimental setting, one form of proliferative responses that is known as lymphopenia-induced homeostatic proliferation (LIP) can be observed. Since LIP is driven by self-pMHC and IL-7, the strength of these signals can be estimated according to proliferative T cell responses. The other form of proliferative responses known as spontaneous proliferation is much stronger than LIP and can be seen in SPF, but not GF, lymphopenic mice adoptively transferred with polyclonal T cells. Spontaneous proliferation of T cells is specific to the gut microbiota and are profoundly reduced in GF lymphopenic mice (Surh and Sprent, 2008; Kim et al., 2018). Interestingly, the levels of LIP in GF *Rag1^-/-^* mice are similar to those seen in SPF *Rag1^-/-^* mice, indicating that gut microbiota does not play a decisive role in the maintenance of peripheral T cells by regulating the IL-7 availability in the secondary lymphoid organs (Kim et al., 2018). Furthermore, absence of commensal microbiota does not lead to the alteration of ILC’s capacity to modulate the availability of IL-7 for conventional T cells (Martin et al., 2017).

The above studies focused on the regulation of T cell maintenance primarily in secondary lymphoid tissues. Considering that ILCs are predominantly tissue-resident immune cells, which are abundantly found in mucosal tissues such as the intestine (Meininger et al., 2020), it is plausible that ILCs can regulate the maintenance of tissue-resident T cells, especially tissue-resident memory CD8^+^ T cells, by modulating the availability of IL-7. Maintenance and survival of tissue-resident memory T cells are dependent on IL-7 (Adachi et al., 2015). These intestinal tissue-resident memory CD8^+^ T cells are mostly derived from conventional CD8αβ T cells in the intestinal epithelium compartment (Konjar et al., 2017). Interestingly, intestinal epithelial cells can produce IL-7 in an inducible manner, which is dependent on microbiota-mediated induction of IFN-γ (Shalapour et al., 2010) (**Figure 1A**). Depletion of gut microbiota by broad spectrum antibiotics treatment or IFN-γ neutralization results in the reduction of IL-7 production by intestinal epithelia.

In addition to the regulation of IL-7 bioavailability by tissue-resident ILCs and intestinal epithelial cells, microbial metabolites can directly influence the generation and maintenance of memory CD8^+^ T cells that are generated upon viral and microbial infection, thereby influencing T cell repertoires systemically or at local tissues such as the intestine (Overacre-Delgoffe and Hand, 2022). Microbial production of SCFAs enhance virus-specific long-lived memory CD8^+^ T cells. Therefore, memory T cell formation is defective in mice absent of commensal microbiota (Overacre-Delgoffe and Hand, 2022). Conversely, high-fiber diets augment memory T cell differentiation. Microbiota-produced butyrate increases the uptake and oxidative phosphorylation of fatty acids in memory CD8^+^ T cells that support their survival (Bachem et al., 2019).

It is known that Treg cells during the initiation of CD8^+^ T cell responses against pathogen infection can shape the fate of activated CD8^+^ T cells by offsetting the pro-effector signals delivered by Ag, costimulatory molecules and pro-inflammatory cytokines (Laidlaw et al., 2016). During T cell priming, the absence of Treg cells hampers the generation of functional memory CD8^+^ T cells (de Goer de Herve et al., 2012). Indeed, IL-10 produced by Treg cells promotes memory CD8^+^ T cell maturation, especially central memory CD8^+^ T cells (Cui et al., 2011; Laidlaw et al., 2015). Considering that SCFAs can promote the induction of colonic Treg cells that are specific to microbial Ag and are fully functional to express IL-10, these Treg cells might be involved in shaping intestinal T cell repertoires by modulating tissue-resident memory T cell generation.

## Shaping intestinal T cell repertoire by gut microbiota

In contrast to internal organs and other mucosal tissues such as the lung, the intestine is continuously exposed to vast amounts of foreign Ags under the steady state, mostly derived from diet and the gut microbiota. Based on the mice raised in conditions that are devoid of both dietary Ags and gut microbiota (“Ag-free” mice), these luminal Ags are shown to profoundly shape intestinal CD4^+^ T cell responses, especially intestinal Treg cells. Intestinal Treg cells consist of heterogenous populations depending on the expression of effector T cell transcription factor (GATA3, RORγt, and Tbet), and their origins (**Figure 1**). Thymic Treg cells express Helios (Thornton et al., 2010) and Neuropilin-1 (Nrp-1) at high levels (Weiss et al., 2012), while pTreg cells in the intestine express Helios and Nrp-1 at low levels and are further divided into two subsets depending on RORγt expression (Kim et al., 2016; Tanoue et al., 2016). A broad spectrum of microbial species is capable of inducing intestinal Foxp3^+^ Treg cells, in particular, Nrp-1^lo^ RORγt^+^ Treg cells in both small intestine and colon (Atarashi et al., 2011; Geuking et al., 2011; Atarashi et al., 2013; Verma et al., 2018) (**Figures 1A, B**). Deprivation of gut microbiota as seen in GF and antibiotics-treated mice results in the reduction of pTreg cells expressing RORγt (Ohnmacht et al., 2015; Sefik et al., 2015; Kim et al., 2016). However, mono-association of various microbial species sufficiently restores colonic Treg cells in GF mice (Sefik et al., 2015). In addition, Ag-free mice display severely reduced numbers of CD4^+^ T cells and Nrp1^-^ pTreg cells, including both RORγt^+^ and RORγt^-^ pTreg cells in the small intestine (Kim et al., 2016). This indicates that dietary Ags also profoundly shape intestinal CD4^+^ T cell responses, in particular RORγt^-^ pTreg cells (**Figure 1A**). Induction of intestinal pTreg cells by the gut microbiota is mediated by various mechanisms (**Figure 1B**). Short chain fatty acids, such as butyrate, acetate and propionate, produced by microbial fermentation of dietary fibers can induce colonic Treg cells (Arpaia et al., 2013; Furusawa et al., 2013; Smith et al., 2013; Marino et al., 2017). Microbial secondary metabolites of bile acids induce the generation of colonic Treg cells as well (Hang et al., 2019; Campbell et al., 2020). In addition, microbial components such as polysaccharide A of *Bacteroides flagilis* and cell surface polysaccharides of *Bifidobacterium bifidum*, effectively induce colonic Treg cells that are fully functional to suppress harmful colitogenic T cell responses (Round and Mazmanian, 2010; Verma et al., 2018).

The above experimental strategies to elucidate the role of the gut microbiota in shaping intestinal CD4^+^ T cell responses do not decisively determine the induction of cognate Ag-specific T cell responses against microbial or dietary Ags. To examine intestinal CD4^+^ T cell responses against the gut microbiota in Ag-specific manner, TCR-Tg cell lines specific to microbial Ags from various microbial species and microbial peptide-loaded MHC tetramer have been intensively used (**Table 1**). Microbes belonging to the *Clostridiales*, SFB, Helicobacter spp., *Akkermansia miciniphila* and *Bacteroides thethaiotaomicron* have been described to induce the activation of either adoptively transferred cognate Ag-specific TCR-Tg cells or microbial pMHC tetramer^+^ T cells (Russler-Germain et al., 2017). Based on these experimental approaches, it has been clearly shown that microbial species distinctly induces the differentiation of Treg cells and effector CD4^+^ T cells. Under the steady state condition, intestinal CD4^+^ T cell responses are well-balanced with aforementioned pTreg cells and pro-inflammatory T cells, but during inflammatory setting, such as experimental inflammatory bowel disease, colonic CD4^+^ T cell responses are skewed to proinflammatory T cells producing IFN-γ and IL-17. SFB favors small intestinal Th17 T cell responses by inducing serum amyloid protein A that promote Th17 cell differentiation (Lee et al., 2020b). *A. muciniphila* efficiently induce CD4^+^ T follicular helper cells (Ansaldo et al., 2019). The capacity of individual microbial species to modulate CD4^+^ T cell differentiation depends on the unique production of microbial metabolites such as SCFA and secondary bile acids, immune modulating microbial constituents including specific microbe-associated molecular patterns, the extent of innate immune responses that governs adaptive T cell responses, and also the immunological context. *H. hepaticus* strongly induces cognate Ag-specific RORγt^+^ Foxp3^-^ Th17 responses in *IL-10^-/-^* mice or mice treated with IL-10 blockades, thereby inducing the experimental colitis. However, under the steady state condition, *H. hepaticus* induces RORγt^+^ Foxp3^+^ Treg cells (Xu et al., 2018).

**Table 1:** Methods to examine the regulation of T cell repertoires by gut microbiota.

Methods	Advantages	Disadvantages
TCR-Tg cell transfer	- Easier identification of Ag-specific T cells by using congenic marker - Precise determination of Ag-specific T cell proliferation (CFSE or CTV labeled donor T cells) - Examination of the interaction between Ag-specific T cells and other immune cells (i.e., B cells) - Single TCR specificity	- Not reflect the physiological frequency of cognate Ag specific T cells - Intra-clonal competition distorts natural Ag-specific T cell responses against microbiota. - Limited use depending on HLA types
**pMHC** Tetramer staining	- Direct examination and quantification of Ag-specific T cell frequency under steady state and inflammatory settings - Examination of physiological Ag-specific T cell responses	- Relatively lower affinity between pMHC tetramer and TCR (limitation of visualization in flow cytometry) - Limited use depending on HLA types - Broad TCR specificities within pMHC tetramer^+^ T cells
Bulk TCR gene sequencing	- Less expensive than single cell based TCR gene sequencing - Relatively bigger sample size	- Lack of information on the TCR diversity created by TCR chain pairing* - Limited association between TCR clonality and T cell subset/function (cytokine, transcription factor expression)**
Single cell-based TCR gene sequencing	- Strong association with TCR clonality and T cell subset/function - Possible examination of the TCR diversity created by TCR chain pairing - Combination with single cell-based transcriptomics & other -omics	- Relatively expensive and limitation on sample size (typically less than 2-3 x 10^4^) - Requirement of complex analytical methods


*Assessment of TCR chain paring is not required in case of bulk TCR gene sequencing based on TCRα or TCRβ-restricted mice., **addressed by TCR sequencing on T cells sorted based on the transcription factor or cytokine reporters.

Examination of cognate T cell responses against microbial Ags by using adoptively transferred TCR-Tg cells has a limitation mainly caused by intraclonal competition (**Table 1**). As the number of adoptively transferred TCR-Tg cells is typically much higher than the number of cognate Ag-specific T cells, donor T cell activation and proliferation do not reflect natural immune responses toward the gut microbiota. Hence, studies based on pMHC tetramer staining can verify the induction of microbial Ag-specific T cell responses in the intestine in a more physiologically relevant manner (**Table 1**). pMHC tetramers also enable the examination of the presence of microbial Ag-specific T cells in other extra-intestinal sites such as the thymus (Zegarra-Ruiz et al., 2021). However, considering that pMHC tetramer^+^ T cells can have broad TCR specificity and that T cell epitopes can govern the fate of CD4^+^ T cell differentiation (Hwang et al., 2020), it remains elusive whether the TCR specificities of pMHC tetramer^+^ T cells can be different depending on the CD4^+^ T cell subset and the immunological context.

Advances in next-generation sequencing (NGS) enables the direct examination of TCR repertoires of T cells responding to the gut microbiota [for a detailed review of sequencing-based TCR repertoire analysis, see Pai and Satpathy (2021)]. During steady state and disease conditions such as experimental colitis, TCR diversity and clonality of CD4^+^ T cells responding to gut microbiota have been determined. For example, by using TCRβ-transgenic mice (TCli TCRβ-Tg x TCRα^+/−^ mice possessing a fixed TCRβ chain that recognized human CLIP and variable TCRα alleles with no chance of allelic inclusion), TCR usages of colonic Treg cells could be compared with those of colonic effector CD4^+^ T cells and Treg cells in other peripheral tissues such as spleen, where the majority of Treg cells represents thymic Treg cells (**Table 1**) (Lathrop et al., 2011). Interestingly, the TCR repertoires of colonic Treg cells rarely overlap with those of colonic effector CD4^+^ T cells. Even in inflammatory settings such as in the T cell-transfer induced experimental colitis model, dominant TCRα clonotypes were shared between IFN-γ- and IL-17-producing but not Foxp3^+^ Treg cells (Muschaweck et al., 2021). One of possible explanation is that distinct microbial species differentially induce Treg and effector T cells, presumably due to the production of unique immune-modulating microbial signals and also due to the induction of unique innate signals. It was shown, for example, that broad spectrum of microbial species, such as altered Schaedler flora (a consortium of eight anaerobic microbial species) and *Bifidobacterium bifidum*, specifically induces intestinal Treg cells but not effector T cells, such as Th17 cells upon the colonization into GF mice (Geuking et al., 2011; Sefik et al., 2015; Verma et al., 2018). However, TCR-Tg CD4^+^ T cells, i.e., Flagellin-specific CD4^+^ Tg (Cbir) T cells, induce experimental colitis upon the transfer into lymphopenic mice, while the administration of Treg-inducing *Bifidobacterium bifidum* ameliorates the colitis by inducing the generation of Cbir Treg cells (Verma et al., 2018). Given that *B. bifidum* does not express flagellin, not only the TCR clonality but also the environmental cues conditioned by *B. bifidum*, are critical for determining the fate of colonic Ag-specific CD4^+^ T cells. Consistently, naïve TCR-Tg cells originated from colonic Treg cells can be differentiated into pathogenic effector CD4^+^ T cells upon the adoptive transfer into lymphopenic mice (Lathrop et al., 2011).

It was shown that TCR repertoires of colonic Treg cells are distinct from Treg cells in other locations. Some TCR originating from colonic Treg cells did not induce the selection of thymic Treg cells upon the TCR gene transduction into thymocytes (Lathrop et al., 2011), emphasizing the presence of Treg cells generated from *de novo* differentiation of naïve CD4^+^ T cells. However, contradictory results have been reported showing that the TCR repertoires of colonic Treg cells markedly overlapped with those of thymic Treg cells (Cebula et al., 2013). Although the reasons for this contradiction were not clearly understood, the presence of entro-thymic delivery of microbial Ags and thymic output of microbial Ag-specific T cells might result in the expansion of thymic Treg cells in the colon (Zegarra-Ruiz et al., 2021). Furthermore, it seems possible that self-Ag specific thymic Treg cells cross-reactive to microbial Ags can expand in the colon. Regardless of the origin of colonic Treg cells, TCR diversity of colonic Treg cells is important for intestinal homeostasis and prevention of colonic inflammation. For example, mice genetically modified to possess restricted TCR repertoires developed spontaneous colitis mediated by colonic Th17 cells due to the reduction of Helios^-^ pTreg cells. Transfer of wildtype Treg cells with intact TCR repertoires could prevent spontaneous colitis (Nishio et al., 2015).

## Role of microbe-specific T cells cross-reactive to self-antigens and neoantigens in autoimmune disease and tumor progression

**Figure 2:**
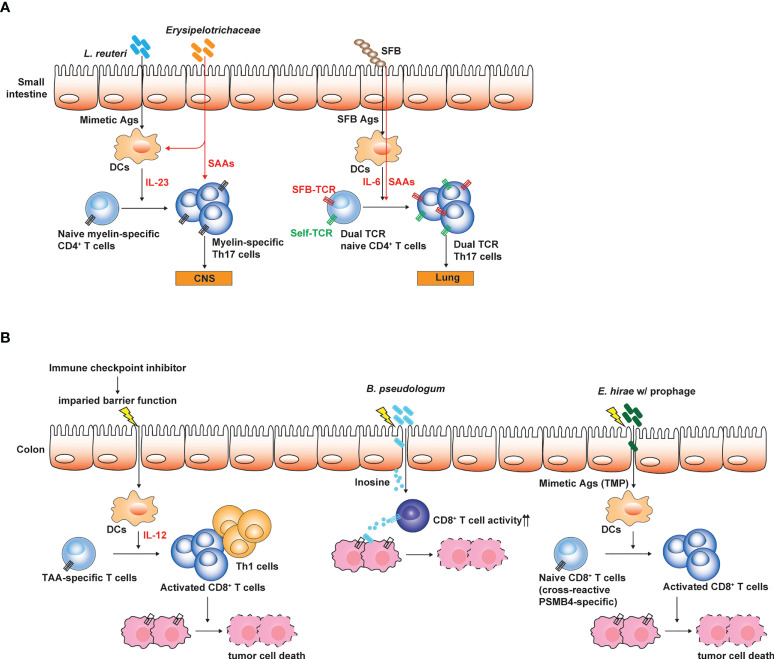
Influences of gut microbiota on the pathogenesis of autoimmune disease and tumors. **(A)** Small intestinal Th17 responses are linked to the susceptibility to Th17-mediated autoimmune disease such as EAE. Antigenic mimicry between microbial Ags and self-Ags is one of the mechanisms through which gut microbiota influences the induction and the pathogenesis of Th17-mediated autoimmune diseases. Myelin-specific CD4^+^ T cells expand by microbial mimetic Ags and are conditioned to differentiate into Th17 cells. Alternatively, T cells expressing dual TCRs specific to both SFB Ag and self-Ag proliferate and differentiate into Th17 cells, and then induce autoimmunity in extra-intestinal tissues. **(B)** Gut microbiota determines the responsiveness to ICI treatment. Weakening intestinal barrier functions by the ICI treatment leads to the translocation of gut microbes and enables gut microbes to promote Th1 and cytotoxic T cell responses against TAA by conditioning intestinal DCs. Bifidobacterium spp. enhance the cytotoxic activity of TAA-specific CD8^+^ T cells by producing inosine. *E hirae* produce mimetic Ag (TMP) which can proliferate cross-reactive TAA (PSMB4)-specific CD8^+^ T cells, thereby enhancing the efficacy of ICI treatment. The figure was created with Adobe illustrator.

Considering that the genomic diversity of commensal microbiota is 100-fold greater than that of the host (Qin et al., 2010), the activation of self Ag-specific CD4^+^ T cells which drive autoimmune diseases can be induced by cross-reactive microbial Ags produced by the gut microbiota (Sprouse et al., 2019). Furthermore, a single TCR can be highly cross-reactive and recognize 10^4^ - 10^6^ different MHC-bound peptides (Lee et al., 2020a). Experimental evidence suggests that antigenic mimicry can be a mechanistic link for the association with the dysbiosis of gut microbiota and various immune disorders (Sprouse et al., 2019). In autoimmune uveitis caused by the breakdown of blood-retinal barrier and the activation of retina-specific Th17 cells, retina-specific T cells can migrate into the intestine and can be activated by non-cognate Ags produced by the gut microbiota (Horai et al., 2015). Antibiotics treatment significantly suppressed the induction of autoimmune uveitis. As aforementioned, GF mice are resistant to EAE relative to SPF mice that possess Th17 cells in the small intestine. Hiroshi Ohno group showed that myelin oligodendrocyte glycoprotein (MOG)-specific autoreactive T cells can migrate into the small intestine and can be activated by non-cognate microbial Ags. MOG-specific autoreactive T cells can proliferate in response to *Lactobacillus reuteri* that expresses cross-reactive microbial Ags and are also conditioned to differentiate into Th17 cells by the action of the other microbial species in Erysipelotrichaceae family (Miyauchi et al., 2020) (**Figure 2A**). In this regard, GF mice colonized with these two microbial species showed more severe EAE symptoms than GF mice or mono-colonized mice. The gut microbiota through the antigenic mimicry also drives autoimmune diseases induced by autoreactive CD8^+^ T cells (Tai et al., 2016). Based on the NOD mice carrying diabetogenic CD8^+^ T cells specific to islet-specific glucose-6-phosphatase catalytic subunit–related protein (IGRP), IGRP-specific T cells can be activated by microbial Ags produced by *Fusobacteria*. In addition, the failure of allelic exclusion during the T cell development in the thymus permit the circulation of T cells with dual TCRs, each specific to self and microbial Ags (Bradley et al., 2017). Therefore, the gut microbiota can induce the activation of dual Ag-specific T cells, consequently promoting the autoimmune diseases. For example, SFB administration exacerbated Th17-mediated autoimmunity in the lung. Activation of autoreactive Th17 cells did not rely on antigenic mimicry or bystander activation. SFB expand Th17 cells expressing dual TCRs specific for SFB epitope and self-Ag (**Figure 2A**). Migration of autoreactive T cells cross-reactive to microbial Ag into the intestine precedes the conditioning in the intestine for the differentiation into Th17 cells. Interestingly, IL-7 signaling can promote gut homing receptor (α4β7) expression in T cells during lymphopenic conditions (Cimbro et al., 2012). IL-7 signaling not only promotes T cell survival and maintenance in the periphery, but also possibly endows T cells with the capacity of migrating into the intestine.

The gut microbiota determines the responsiveness of tumor patients to immune checkpoint inhibitor (ICI) treatment that reinvigorates the tumor associated Ag-specific exhausted CD8^+^ T cells. The microbial composition of the gut microbiota in tumor patients responding to ICI treatment was different to that of non-responders. Furthermore, the transfer of fecal microbiota from responders into tumor-bearing GF mice increased anti-tumor immunity, thereby profoundly reducing the tumor burdens (Gopalakrishnan et al., 2018; Matson et al., 2018; Routy et al., 2018). Additionally, transplantation of fecal microbiota from the responders or healthy individuals made the non-responders become responsive to ICI treatment (Baruch et al., 2021; Davar et al., 2021). Interestingly, ICI-induced colitis, a severe form of gastrointestinal symptoms in patients receiving ICI, is closely associated with IFN-γ producing CD8^+^ tissue resident memory T cells in the intestine (Sasson et al., 2021).

How the gut microbiota enhances the anti-tumor immunotherapy largely remains unclear and is a key research topic that attracts great interest. Emerging evidence revealed that the gut microbiota can modulate anti-tumor immunity through various mechanisms (Sepich-Poore et al., 2021) (**Figure 2B**). For example, certain microbes with anti-tumor effects, such as *Bacteroides thetaiotaomicron* and *B. fragilis* can activate dendritic cells through Toll-like receptor (TLR)-4 signaling and promote Th1 and cytotoxic CD8^+^ T cell responses helpful for the tumor immunosurveillance and eradication (Vetizou et al., 2015). *Bifiodbacterium pseudologum* can produce inosine, which enhance the cytotoxic activity of CD8^+^ T cells by agonizing adenosine 2A receptor signaling in T cells (Mager et al., 2020). Enhancement of anti-tumor immunotherapy by the gut microbiota is typically associated with gut barrier dysfunction and the translocation of gut microbes, which can be induced by total body irradiation used for adoptive CAR T cell transfer (Uribe-Herranz et al., 2020) or ICI treatment (Mager et al., 2020). Anti-CTLA-4 treatment weakens the intestinal barrier and permits microbial translocation and systemic circulation of microbe-derived inosine (Mager et al., 2020). Indeed, better outcomes of ICI treatment were associated with gastrointestinal immune-related adverse events (Zhou et al., 2020).

As seen by the role of gut microbiota in Th17-mediated autoimmune diseases, gut microbes represent an Ag source which stimulate cross-reactive tumor-associated Ag (TAA)-specific CD8^+^ T cells. For example, Laurence Zitvogel group showed that *Enterococcus hirae* haboring prophage expressing tail length tape measure proteins (TMPs) in its genome mount CD8^+^ T cell responses against an oncogenic driver, proteosome subunit beta type-4 (PSMB4) through the antigenic mimicry (Fluckiger et al., 2020) (**Figure 2B**). In this regard, *E. hirae* administration enhances anti-tumor CD8^+^ T cell responses upon the treatment with cyclophosphamide or anti-PD-1 antibodies. Recently, based on Blast search for homology between TAA and Ags produced by gut microbiota (belonging to Firmicutes and Bacteroides phyla) combined with epitope prediction analysis considering HLA polymorphism, it was proposed that antigenic mimicry between TAA and microbial Ags produced by gut microbiota can be much broader and highly possible to influence anti-tumor immunity and tumor progression through the activation of TAA-specific CD8^+^ T cells by cross-reactive microbial Ags (Ragone et al., 2022).

## Conclusion and perspectives

Research on the role of the gut microbiota in health and diseases has progressed to establish the causal relationship between the gut microbiota and the disease phenotypes (Finlay et al., 2020). Furthermore, recent findings have demonstrated the feasibility of microbiota-based therapeutics for treating various intractable diseases. Examination of the interaction between the host immune system and the gut microbiota in Ag-specific manner improves our understanding on the role of gut microbiota in shaping the host immune system in health and disease and provide a guidance for developing effective microbiome-based therapeutics, i.e., live biotherapeutic products (LBPs). For example, antigenic mimicry seems to be a principal mechanism through which the gut microbiota enhances anti-tumor immunotherapy. In this regard, a microbial consortium, rather than a single LBP, is more suitable for treating tumor patients, especially non-responder to ICI treatment by increasing the chance of TAA-specific CD8^+^ T cell expansion and activation. Furthermore, based on the bioinformatics and computational analysis to reveal the mimicry between TAAs and microbial Ags in the gut microbiota, microbial consortium can be rationally designed to consist of microbes potentially inducing TAA-specific CD8^+^ T cell responses through antigenic mimicry. This strategy can be further improved by incorporating single cell-based or bulk TCR repertoire analysis on tumor-infiltrating T cells and TCR sequence-based prediction of peptide-MHC specificity that also considers HLA haplotypes and the structure of MHC peptide binding grooves (Montemurro et al., 2021).

It seems that T cells involved in extra-intestinal diseases are localized and conditioned in the intestine through the *in situ* activation of T cells by antigenic mimicry or through the gut homing of T cells from extra-intestinal sites. Considering that intestinal T cell responses against the gut microbiota is typically skewed to generate immune tolerance and containment of microbial translocation, proper conditioning of TAA-specific CD8^+^ T cells by gut microbiota in the intestine is required for their expansion and activation, and also for the enhancement of anti-tumor immunotherapy. This can be governed not only by the unique immune-modulating activity of therapeutic microbes but also depends on the immunological context, i.e., inflammatory settings, that can be created in part by the ICI treatment. Evidence showed that T cells with unique TCR recognizing microbial Ags can be differentiated into pathogenic T cells or regulatory T cells depending on the immunological context. Studies on experimental colitis models showed that the re-direction of microbe-specific T cells into Treg cells, but not into pathogenic T cells in an inflammatory setting, is possible by administrating microbial species promoting Treg cell generation. In this regard, microbes that condition the intestinal environment to be more tolerogenic are promising candidates for microbiome-based therapeutics for treating autoimmune diseases and inflammatory bowel diseases.

Currently, mapping the functional landscape of TCR repertoires in extra-intestinal diseases and TCR repertoire analyses to examine the importance of antigenic mimicry between self-Ags/tumor neoantigens and microbial Ags in autoimmune diseases and also in anti-tumor immunotherapy are largely limited. Mapping the functional landscape and comparison of TCR repertoires in the intestine and extra-intestinal tissues or tumor sites is promising to get an important insight on the regulation of the disease pathogenesis by the gut microbiota and also to develop effective microbiome-based therapeutics.

## Author contributions

The author confirms being the sole contributor of this work and has approved it for publication.

## Funding

This work was supported by the BK21 FOUR funded by the Ministry of Education, Korea, and also by grants from the National Research Foundation (NRF) of Korea (NRF-2020M3A9G3080282 and NRF-2020R1A2C1008459).

## Conflict of interest

The author declares that the research was conducted in the absence of any commercial or financial relationships that could be construed as a potential conflict of interest.

## Publisher’s note

All claims expressed in this article are solely those of the authors and do not necessarily represent those of their affiliated organizations, or those of the publisher, the editors and the reviewers. Any product that may be evaluated in this article, or claim that may be made by its manufacturer, is not guaranteed or endorsed by the publisher.
